# Prevalence and correlates of probable common mental disorders in a population with high prevalence of HIV in Zimbabwe

**DOI:** 10.1186/s12888-016-0764-2

**Published:** 2016-02-29

**Authors:** Dixon Chibanda, Frances Cowan, Lorna Gibson, Helen A. Weiss, Crick Lund

**Affiliations:** University of Zimbabwe, College of Health Sciences, Mazowe Street, P.O Box A178, Harare, Zimbabwe; University College London, Gower Street, London, WC1E6BT UK; MRC Tropical Epidemiology Group, London School of Hygiene and Tropical Medicine, Keppel Street, London, WC1E7HT UK; Department of Psychiatry and Mental Health, University of Cape Town, Alan J Flisher Center for Public Mental Health, Cape Town, South Africa

**Keywords:** Common mental disorders, Depression, HIV, Highly active antiretroviral therapy

## Abstract

**Background:**

In 2014 close to 10 million people living with HIV (PLWH) in sub-Saharan Africa were on highly active anti-retroviral therapy (HAART). The incidence of non-communicable diseases has increased markedly in PLWH as mortality is reduced due to use of HAART. Common mental disorders (CMD) are highly prevalent in PLWH. We aimed to determine factors associated with probable CMD and depression, assessed by 2 locally validated screening tools in a population with high prevalence of HIV in Harare, Zimbabwe.

**Methods:**

We carried out a cross-sectional survey of a systematic random sample of patients utilizing the largest primary health care facility in Harare. Adults aged ≥18 years attending over a 2-week period were eligible, excluding those who were critically ill or unable to give written informed consent. Two locally validated screening tools the Shona symptom questionnaire (SSQ-14) and the Patient Health Questionnaire (PHQ-9) were administered by trained research assistants to identify probable CMD and depression.

**Results:**

Of the 264 participants, 165 (62.5 %) were PLWH, and 92 % of these were on HAART. The prevalence of probable CMD (SSQ14 > = 9) and depression (PHQ9 > = 11) were higher among people living with HIV than among those without HIV (67.9 and 68.5 % vs 51.4 and 47.2 % respectively). Multivariable analysis showed female gender and recent negative life events to be associated with probable CMD and depression among PLWH (gender: OR = 2.32 95 % CI:1.07–5.05; negative life events: OR = 4.14; 95 % CI 1.17–14.49) and with depression (gender: OR = 1.84 95 % CI:0.85–4.02; negative life events: OR = 4.93.; 95 % CI 1.31–18.50)

**Conclusion:**

Elevated scores on self-report measures for CMD and depression are highly prevalent in this high HIV prevalence population. There is need to integrate packages of care for CMD and depression in existing primary health care programs for HIV/AIDS.

## Background

Common mental disorders (CMD) affect people across the world with a global lifetime prevalence of 29 % [1]. Presenting as a mixture of somatic, anxiety, and depressive symptoms [2], CMD increase the risk of developing both non-communicable and communicable diseases [3]. Amongst people living with HIV (PLWH), CMD are a leading cause of disability [4–7] and are known to hasten HIV disease progression [8], particularly in LMIC [4, 6, 9] where low levels of CMD detection at primary health care level [10] are confounded by a large treatment gap for CMD [11, 12]. In sub-Saharan Africa it is estimated that there is one psychologist for every 2.5 million people, one mental health nurse for every million people and one psychiatrist for every 2 million people [13]. In contrast, in high income countries the ratio of psychiatrists to the population is estimated to be 1:10000 [14].

In recent years the development of interventions that emphasize task-shifting as a way to address the treatment gap for CMD has gained recognition [15–18] with specific structured packages being developed and evaluated [19–21]. The key elements of task-shifting involve the delegation of responsibilities to lower level cadres, often non-professionals, to deliver services while the more qualified professionals provide support and supervision [22]. However, use of targeted task-shifting psychological interventions to address CMD in PLWH requires a thorough understanding of factors associated with CMD in this population [4, 23], due partially to the need for greater monitoring and supervision of work delivered by non-specialists [24–26].

Much is known about factors associated with CMD in non-HIV populations in LMIC [27–31], contrasting with sparse data on CMD among PLWH [6]. In four LMIC, namely India, Zimbabwe, Chile and Brazil, female gender, poverty and recent life events were found to be common correlates of CMD in non-HIV populations [29]. In other parts of the world, correlates of CMD in PLWH include death of a significant other [32], family history of mental illness, negative coping style, alcohol dependency, food insecurity [33] and recent negative life events [34, 35]. Other studies from poor resource settings have found that CMD in PLWH is correlated with trauma, posttraumatic stress disorder (PTSD), stigma and social barriers such as peoples’ attitudes towards disabilities, physical and organizational obstacles, however, these differences appear to be due to a difference in the study setting and study population [36–39].

Early studies of CMD in PLWH in Zimbabwe were based on antiretroviral (ART) -naïve populations using the Shona Symptom Questionnaire (SSQ-14) [40] as the main outcome instrument. Findings were inconsistent, with correlates of CMD including being female, having a male partner who was older by 10 or more years, multi-parity (having borne a number of children), and negative life events [34, 41, 42].

The aim of this study was to determine the prevalence and correlates of probable CMD in a primary health care setting with a high prevalence of HIV, and to compare these correlates of CMD by HIV status. The study was part of the validation of screening tools for CMD in a population with a high prevalence of HIV, which was preparatory work for a randomized controlled trial of a psychological intervention to reduce depression. The trial used the SSQ-14 and the 9-item Patient Health Questionnaire (PHQ-9) as primary and secondary outcome measures respectively. Establishing the prevalence of CMD among primary health care patients helped determine the possible duration of recruitment of participants for the trial, and analysis of correlates of CMD contributed towards the development of appropriate interventions [43].

## Methods

### Study setting and population

The study was carried out in the largest HIV primary health care clinic in Harare where up to 200 PLWH are seen daily. The primary health facility, which runs a daily HIV clinic, focuses mainly on prescribing ART and treating other HIV-related conditions, whilst also catering for both adults and children with non-HIV related conditions. All adults aged ≥18 years presenting to the clinic during the 2-week study period from 14th-30th October 2013 were eligible for recruitment. Patients were excluded if they were critically ill (being physically incapacitated), had a psychotic episode at presentation, or were unable to comprehend the consenting procedure or declined to give written informed consent.

### Study procedure

Study personnel (four research assistants, six LHWs and four psychiatrists) attended a 2-week training using a guide initially developed by the author (DC). The research assistants were trained on data collection methods using the socio-demographic forms and the screening tools. While the project coordinators were trained in screening of participants to minimize bias. The psychiatrists were trained in the use of the structured clinical interview (SCID-IV) through a discussion forum led by DC which involved going through the diagnostic criteria, building consensus on how to manage clinically severe cases during the validation, and procedures for ensuring fidelity. The referral pathways for participants meeting criteria for major depression and other acute medical conditions was that they should be seen by the medical officer first, for assessment, before being referred to a tertiary psychiatric facility if needed. A 2-day pilot of the full study procedure was carried out after training of the entire study team.

### Ethical considerations

Ethical approval was obtained from the Medical Research Council of Zimbabwe (MRCZ/A/1732), and the Human Research Ethics Committee of the Faculty of Health Sciences, University of Cape Town (HREC Ref: 090/2014) and the London School of Hygiene and Tropical Medicine (Ref 8457), and written informed consent was sought from all participants in accordance with good clinical practice. All participants requiring immediate attention due to severe CMD were referred to an existing service for people with psychological distress based on problem solving therapy running at the clinic [44].

### Recruitment and sample size

The clinic register of clients attending the clinic over the study period was used as the sampling frame. Based on this sampling frame, computer generated random numbers were used to select participants waiting to be attended to at the study site: random numbers were allocated to patients based on their position in the clinic queue while waiting to be attended to by the clinic nurse. This exercise was carried out while all the patients were in the waiting room waiting to be triaged to nursing evaluation. All those selected were sensitized, informed briefly about the study before they were asked if they were willing to participate. All interested were then given further details of the study and written consent was sought from eligible adults, while those not meeting the criteria outlined above were excluded at this point. After obtaining written consent the socio-demographic questionnaire and study tools which included the SSQ-14 [40] and the PHQ-9 [45] were administered by data collectors. It took approximately 15–20 min to administer tools to each participant.

The sample size (*N* = 264) was chosen to provide good precision for the primary aim of the validation study, to estimate sensitivity and specificity for screening tools against the gold standard (SCID), allowing for stratification by HIV status. This sample size enables us to estimate a prevalence of CMD of 28 % with good precision (95 % CI 22-34 %), and to have 80 % power to detect odds ratio of 2.5 for an exposure which is 50 % prevalent in the controls.

### Study measures

Socio-demographic variables were measured using an adapted locally developed questionnaire previously used in similar studies where variables such as unemployment, recent negative life events, poverty and female gender had been found to be associated with CMD [30, 31, 34, 46]. Participants were asked about recent negative life events. The list of possible negative life events was based on a previous study [34] and focus group discussions with both the lay health workers and participants utilizing a local CMD based intervention [44]. These included life events such as death in the family, physical assault, sexual assault, forced eviction, an HIV diagnosis, and an illness resulting in admission to a tertiary hospital of either the participant or an immediate family member.

### Screening tools

The Shona Symptom Questionnaire (SSQ14) was used as the primary outcome measure for CMD screening. The SSQ14 is a locally developed 14-item screening tool validated using exemplary cross cultural methods [40]). It consists of 14 dichotomous questions based on how an individual has been feeling in the past week. It has been used previously in epidemiological studies in Zimbabwe [41, 44, 47] and has sensitivity of 96 %, specificity of 83 %, and positive predictive value and negative predictive value of 66 and 83 % respectively, using a cut-off of ≥8 [40]. In this study population, we found an optimal cut-off score of > = 9 in a validation exercise carried out by senior doctors in the Department of Psychiatry using the structured clinical interview (SCID-IV) as the gold standard (results not published - sensitivity and specificity at cut-off of > = 9 were 85.9 and 70.4 % respectively).

The 9-item Patient Health Questionnaire (PHQ-9) [45] was the instrument used to screen for probable depression. The PHQ-9 is amongst the most commonly used screening instrument for depression in LMIC [48], and uses a Likert scale giving a score ranging from 0 to a maximum of 27 with each of the 9 items giving a response ranging from: Not at all (0); Several days (1); More than half the days (2); and Nearly every day (3), and a higher score indicating more severe depression [45]. The PHQ-9 was validated during the formative stage of the current study using the structured clinical interview of the diagnostic statistical manual (SCID-IV), and showed a sensitivity of 84.6 % for depression and specificity of 68.7 % against the SCID at a cut-off of > =11 (results not published). We therefore used this validated cut-off score of 11 and above for moderate depression.

### Statistical analysis

Data were entered directly into the study desk-top computer by a data entry clerk using a predesigned data entry program containing automated range checks, and data cleaning was carried out at the end of each day*.* Data were transferred to STATA version 13.0 for analysis. Analysis was based on outcome measures of the SSQ-14 for CMD and PHQ-9 for depression. Following tests for effect modification of HIV status and factors associated with CMD, results were presented stratified by HIV status. Socio-demographic variables of the two groups (cases vs. non-cases) meeting SSQ-14 and PHQ-9 criteria for CMD and depression respectively were initially compared to establish differences. Variables with *p* < 0.15 on univariate logistic regression analyses were included in multivariable regression, to estimate adjusted odds ratios (OR) and 95 % confidence intervals (CI).

## Results

### Characteristics of study participants

A total of 332 people were approached during the study period. Of these 297 (89 %) were eligible, and 264 (89 %) of those who were eligible gave consent to take part. Of the 264 included in the study, 208 (79 %) were female and 155 (59 %) were married. The majority (237; 89 %) reported experiencing a negative life event in the 6-month period before the study. HIV was highly prevalent, with 165 (62.5 %) reported as living with HIV according to self-reports confirmed with clinic HIV test records, while 72 (27 %) were HIV negative. A total of 27 (10 %) were unaware of their HIV status because they had never been tested. All participants approached were forthcoming with their HIV status. Of the 165 who were confirmed HIV positive, almost all (92 %) were on HAART, with most of these (85 %) on HAART for more than 6 months.

Table 1 show characteristics of the study population by HIV status. People living with HIV were more likely to be female (75.2.% vs 24.8 %; *p* = 0.008), older (47.2 % vs 26.4 % aged ≥40 years; *p* = 0.009), and divorced/widowed (25.5 % vs 5.6 %; *p* = 0.001).

**Table 1:** Characteristics of study participants by HIV status^a^

Characteristic	HIV positive (*n* = 165)		HIV negative (*n* = 72)		*p*-value
	N	%	N	%	
Gender					0.008
Male	41	24.8 %	7	9.7 %	
Female	124	75.2 %	65	90.3 %	
Age group					0.009
< 30	26	16.1 %	22	32.4 %	
30–39	59	36.6 %	28	41.2 %	
40–49	56	34.8 %	12	17.6 %	
50+	20	12.4 %	6	8.8 %	
Marital status					0.001
Married/steady	87	52.7 %	57	79.2 %	
Divorced/widowed	42	25.5 %	4	5.6 %	
Single	36	21.8 %	11	15.3 %	
Education					0.21
Secondary or more	127	77.0 %	58	80.6 %	
Primary or less	38	23.0 %	14	19.4 %	
Current employment status					0.78
Unemployed	69	41.8 %	33	45.8 %	
Permanent FT or PT	16	9.7 %	7	9.7 %	
Casual/self-employed	80	48.5 %	32	44.4 %	
Main income source					0.20
Own business/salary	96	58.2 %	35	48.6 %	
Partner/family	58	35.2 %	34	47.2 %	
No income	11	6.7 %	3	4.2 %	
Suffer from chronic illness					0.49
No	86	52.1 %	41	56.9 %	
Yes	79	47.9 %	31	43.1 %	
Reason for clinic visit					0.02
Routine/family/antenatal	63	38.2 %	39	54.2 %	
Other reason	102	61.8 %	33	45.8 %	
Negative life events in last six months					0.15
No	13	7.9 %	10	13.9 %	
Yes	152	92.1 %	62	86.1 %	
SSQ ≥ 9					0.02
No	53	32.1 %	35	48.6 %	
Yes	112	67.9 %	37	51.4 %	
PHQ ≥ 11					0.002
No	52	31.5 %	38	52.8 %	
Yes	113	68.5 %	34	47.2 %	

^a^HIV status unknown (*n* = 27) not included

### Association of probable CMD and depression by HIV status

The prevalence of probable CMD (SSQ14 ≥ 9) and depression (PHQ9 ≥ 11) were higher among people living with HIV than among those without HIV (67.9 and 68.5 % vs 51.4 and 47.2 % respectively; Table 1). On univariable analyses, both probable CMD and depression were associated with being female, and having experienced negative life events (Table 2).

**Table 2:** Characteristics of HIV + participants by SSQ-14 and PHQ-9 scores

	SSQ ≥ 9					PHQ ≥ 11				
			Univariable analysis					Univariable analysis		
	Total N	%	OR	95 % CI	*p*-value	Total N	%	OR	95 % CI	*p*-value
Gender					0.03					0.05
Male	41	53.7 %	1.00	-		41	56.1 %	1.00	-	
Female	124	72.6 %	2.29	(1.10–4.74)		124	72.6 %	2.07	(1.00–4.31)	
Age group					1.00					0.66
< 30	26	69.2 %	1.00	-		26	65.4 %	1.00	-	
30–39	59	67.8 %	0.94	(0.35–2.53)		59	69.5 %	1.21	(0.45–3.21)	
40–49	56	67.9 %	0.94	(0.34–2.56)		56	66.1 %	1.03	(0.39–2.74)	
50+	20	70.0 %	1.04	(0.29–3.69)		20	80.0 %	2.12	(0.54–8.26)	
Marital status					0.75					0.37
Married/steady	87	65.5 %	1.00	-		87	66.7 %	1.00	-	
Divorced/widowed	42	69.0 %	1.17	(0.53–2.59)		42	64.3 %	0.90	(0.42–1.95)	
Single	36	72.2 %	1.37	(0.58–3.21)		36	72.8 %	1.75	(0.71–4.32)	
Education					0.93					0.01
Secondary or more	127	67.7 %	1.00			127	63.8 %	1.00		
Primary or less	38	68.4 %	1.03	(1.45–3.04)		38	84.2 %	3.03	(1.18–7.79)	
Currently working?					0.53					0.86
Unemployed	69	71.0 %	1.00	-		69	69.6 %	1.00	-	
Permanent FT or PT	16	56.3 %	0.52	(0.17–1.60)		16	62.5 %	0.73	(0.23–2.57)	
Casual/self-employed	80	67.5 %	0.85	(0.42–1.71)		80	68.8 %	0.96	(0.48–1.93)	
Main income source					0.51					0.17
Own business/salary	96	65.6 %	1.00	-		96	65.6 %	1.00	-	
Partner/family	58	69.0 %	1.16	(0.58–2.34)		58	69.0 %	1.16	(0.58–2.34)	
No income	11	81.8 %	2.36	(0.48–11.55)		11	90.9 %	5.24	(0.64–42.70)	
Chronic medical condition					0.07					0.33
No	86	61.6 %	1.00	-		86	65.1 %	1.00	-	
Yes	79	74.7 %	1.84	(0.94–3.58)		79	72.2 %	1.39	(0.72–2.69)	
Suffer from other chronic illness					0.75					0.43
No	127	68.5 %	1.00	-		127	66.9 %	1.00	-	
Yes	38	65.8 %	0.88	(0.41–1.91)		38	73.7 %	1.38	(0.61–3.11)	
Negative life events					0.004					0.02
No	13	30.8 %	1.00	-		13	38.5 %	1.00	-	
Yes	152	71.1 %	5.52	(1.62–18.87)		152	71.1 %	3.93	(1.22–12.67)	
ARV medication					0.35					0.12
No	14	78.6 %	1.00	-		14	85.7 %	1.00	-	
Yes	151	66.9 %	0.55	(0.15–2.06)		151	66.9 %	0.34	(0.07–1.56)	
Disclosure to partner					0.78					0.44
No	69	66.7 %	1.00	-		69	65.2 %	1.00	-	
Yes	96	68.8 %	1.10	(0.57–2.13)		96	70.8 %	1.30	(0.67–2.51)	

On multivariable analyses, female gender and negative life events were independently associated with both probable CMD and depression among participants with HIV (Table 3). In addition, there was some evidence that probably CMD was associated with having a chronic medical condition (OR = 1.87, 95 % C I 0.92–3.81) and that depression was associated with having less than secondary education (OR = 3.68, 95 % CI 1.35–10.07). The small number of HIV negative participants reduced the ability to look at associations in this group, but there was some evidence that females were at higher risk of probable CMD (OR = 3.05, 95 % CI 0.51–18.12) and stronger evidence that less than secondary education and negative life events were associated with depression (OR = 5.69, 95 % CI 1.26–25.7 and OR = 9.42, 95 % CI 1.36–65.1, respectively)

**Table 3:** Multivariate analysis of factors associated with probable CMD and depression among participants with HIV

Probable depression CMD (SSQ > = 9)	Adjusted^a^ OR	95 % CI	*p*-value
Gender			0.04
Male	1.00		
Female	2.32	(1.07–5.05)	
Chronic medical condition			0.09
No	1.00		
Yes	1.87	(0.92–3.81)	
Negative life events			0.03
No	1.00		
Yes	4.14	(1.17–14.69)	
Depression (PHQ > = 11)			
Gender			0.12
Male	1.00	-	
Female	1.84	(0.85–4.02)	
Education			0.01
Secondary or more	1.00	-	
Primary or less	3.68	(1.35–10.07)	
Negative life events			0.02
No	1.00	-	
Yes	4.93	(1.31–18.50)	

^a^adjusted for other factors in the table

## Discussion

We found a high prevalence of probable CMD and depression (both over 60 %) among PLWH, underpinning the importance of detecting and addressing common mental disorders in this population. Our results indicate that probable CMD and depression in PLWH, as measured by the SSQ-14 and the PHQ-9, are associated with recent negative life events and female gender. These findings reflect those from non-HIV infected populations [29–31, 49]. In a recent systematic review of screening tools used in LMIC, prevalence of 11 %–55 % was reported, however, none of the studies looked at HIV populations on ART [48]. While the risk of false positive screening results from self-report measures is a concern, such tools can contribute towards identifying those most at risk of CMD in resource-poor settings when validated and used within primary health care facilities [6, 7].

Although we did not measure adherence to HAART in our study population, there is growing evidence of an association of CMD and depression with poor adherence to HAART [8, 50, 51]. There is also evidence suggesting that treatment of depression increases adherence to HAART [50, 52]. Despite being on HAART, PLWH face a magnitude of problems and are twice as likely to suffer from depression when compared to HIV negative matched controls [34, 53, 54]. The findings of our study have implications for some of the newer WHO initiatives such as the Option B+ [55] which advocate for immediate commencement of HAART for HIV infected pregnant women. Over 30 % of women attending the Prevention of Mother to Child Transmission of HIV (PMTCT) program in Zimbabwe have signs of post-natal depression [34], therefore the inclusion of CMD packages of care within these initiatives is critical as this will contribute towards improving outcomes for PLWH.

In LMIC the improvement of HAART access will need to be matched with an equally focused integration of evidence-based CMD and depression care packages [6] because there is a large body of data showing that treating CMD among PLWH improves adherence to HAART and quality of life [56–58]. Both HIV positive and HIV negative women are more likely to be affected by CMD and depression in our setting [29, 34]. Furthermore they are more likely to utilize primary health care facilities than their male counterparts [59]. Therefore focusing on women who are living with HIV at primary health care level, particularly those with negative life events who screen positive on the SSQ-14 and PHQ-9 will strengthen existing HIV related programs such as the PMTCT and the newer Option B+. There is evidence showing the feasibility of using screening tools at primary health care level in Zimbabwe [34, 41, 44, 60] but these tools are not integrated into HIV care programs.

There are several psychological interventions that have been shown to work in LMIC, which could be adapted for use among PLWH [61–64]. However, most of the studies evaluating the effectiveness of these interventions for PLWH have been of poor quality [25]. Therefore there is a need to conduct rigorous formative research to design interventions and evaluate them before they are introduced or integrated into health care facilities in LMIC. Part of this process should involve establishing factors that are associated with CMD and depression, and using this information to determine the content of interventions for specific populations. Our findings support the use of a problem solving therapy approach because of the strong association between negative life events and CMD [65]. Problem solving therapy has shown promising results in earlier pilot studies in Zimbabwe, particularly among participants who have experienced negative life events [44, 61]. It guides participants through a process to list the problems (negative events) that they face, select a problem to tackle based on the client’s priorities, develop a specific and measurable way of addressing the problem and execute an action plan aimed at addressing the problem while motivating and encouraging the participant to be pro-active in the process [44].

There is evidence supporting the use of lay health workers to deliver such interventions for CMD in LMIC [24] with a number of well designed randomized controlled trials suggesting that this is a cost-effective way of addressing the treatment gap for MNS [21, 66–68].

Our study’s main limitation is the lack of data on HIV staging and viral load markers. These factors can be associated with increased risk of developing CMD and depression [69–71]. Although similar high rates of CMD and depression have been reported from other LMIC [6, 72], the use of a clinic-based sample of people predominately visiting the clinic for physical ailments reduces the generalizability of our findings. Furthermore, the use of screening tools without a gold standard to confirm diagnosis may result in the inclusion of participants who are not actually clinically depressed [73]. Nevertheless, our cut-off score was based on a validation study conducted in Zimbabwe and provide an indication of the likely margin of error on these measures. Studies utilizing clinical interviews have found lower rates but these are still higher than those found in non-HIV infected people [7]. In addition, data on the type of HAART being taken by study participants was not available. A number of HAART drugs can increase the risk of psychiatric disorders including depression [74–76]. Although education and employment have been shown in previous studies to be associated with CMD and depression, our study indicates that this was not the case. This may have been due to the homogenous nature of the study population recruited from the same geographical location with similar socio-economic status. The low uptake of male participants is consistent with previous studies carried out in primary health care facilities in the country and other parts of the world [44, 77–79].

## Conclusion

This study highlights, as in previous earlier studies in non-HIV infected populations, the association of female gender and negative life events with CMD and depression. Of note is the finding of high prevalence of these disorders in PLWH on HAART. This highlights the need to introduce interventions within HIV care clinics at primary health care level aimed at addressing this burden, which if left unchecked could negatively impact the gains made in the fight against HIV/AIDS in the last 2 decades. We further recommend the use of a two-stage screening process particularly where resources are scarce to avoid the inclusion of false positives, with the second stage consisting of a diagnostic screen. However, in resource-poor settings with large numbers of LHWs a greater sensitivity and a lower specificity would be recommended to ensure that all possible cases are included. We further recommend that the Option B+ program be administered with a psychological intervention. Further studies are needed to look into the development of multiple stage screening delivered by LHWs.
